# Increased mtDNA mutations with aging promotes amyloid accumulation and brain atrophy in the APP/Ld transgenic mouse model of Alzheimer’s disease

**DOI:** 10.1186/1750-1326-9-16

**Published:** 2014-05-02

**Authors:** Lokesh Kukreja, Gregory C Kujoth, Tomas A Prolla, Fred Van Leuven, Robert Vassar

**Affiliations:** 1 Department of Cell and Molecular Biology, Northwestern University Feinberg School of Medicine, Chicago, IL 60611, USA; 2 Departments of Genetics and Medical Genetics, University of Wisconsin, Madison, WI 53706, USA; 3 Department of Human Genetics, Experimental Genetics Group-LEGTEGG, KU Leuven, Leuven, Belgium

**Keywords:** *PolgA* D257A, APP/Ld, mitochondrial dysfunction, Amyloid, Aβ, Alzheimer’s disease, Brain atrophy, Neurodegeneration, Insulin degrading enzyme

## Abstract

**Background:**

The role of mitochondrial dysfunction has long been implicated in age-related brain pathology, including Alzheimer’s disease (AD). However, the mechanism by which mitochondrial dysfunction may cause neurodegeneration in AD is unclear. To model mitochondrial dysfunction *in vivo*, we utilized mice that harbor a knockin mutation that inactivates the proofreading function of mitochondrial DNA polymerase γ (*PolgA* D257A), so that these mice accumulate mitochondrial DNA mutations with age. *PolgA* D257A mice develop a myriad of mitochondrial bioenergetic defects and physical phenotypes that mimic premature ageing, with subsequent death around one year of age.

**Results:**

We crossed the D257A mice with a well-established transgenic AD mouse model (APP/Ld) that develops amyloid plaques. We hypothesized that mitochondrial dysfunction would affect Aβ synthesis and/or clearance, thus contributing to amyloidogenesis and triggering neurodegeneration. Initially, we discovered that Aβ42 levels along with Aβ42 plaque density were increased in D257A; APP/Ld bigenic mice compared to APP/Ld monogenic mice. Elevated Aβ production was not responsible for increased amyloid pathology, as levels of BACE1, PS1, C99, and C83 were unchanged in D257A; APP/Ld compared to APP/Ld mice. However, the levels of a major Aβ clearance enzyme, insulin degrading enzyme (IDE), were reduced in mice with the D257A mutation, suggesting this as mechanism for increased amyloid load. In the presence of the APP transgene, D257A mice also exhibited significant brain atrophy with apparent cortical thinning but no frank neuron loss. D257A; APP/Ld mice had increased levels of 17 kDa cleaved caspase-3 and p25, both indicative of neurodegeneration. Moreover, D257A; APP/Ld neurons appeared morphologically disrupted, with swollen and vacuolated nuclei.

**Conclusions:**

Overall, our results implicate synergism between the effects of the *PolgA* D257A mutation and Aβ in causing neurodegeneration. These findings provide insight into mechanisms of mitochondrial dysfunction that may contribute to the pathogenesis of AD via decreased clearance of Aβ.

## Background

Alzheimer’s disease (AD) is a progressive neurodegenerative disorder that is the most common cause of dementia in the elderly [1-3]. A major neuropathological feature of AD is the presence of neuritic plaques that are primarily composed of the 42-amino acid β-amyloid (Aβ) peptide, Aβ42. Aβ peptides are endoproteolytically processed from the amyloid-β precursor protein (APP) via sequential cleavages enacted by BACE1 (β-secretase) and γ-secretase [4]. Most of the genetic factors known to increase susceptibility to Alzheimer’s disease increase the levels of Aβ in the brain (reviewed in [3]). Though numerous studies have shown that cerebral accumulation of Aβ plays a critical early role in AD pathogenesis, the underlying mechanism by which Aβ causes neurodegeneration remains unclear.

Mitochondrial dysfunction has been implicated in age-related cognitive decline (reviewed in [5]) and potentially plays a central role in the progression of Alzheimer’s disease. Pyramidal neurons, which require large amounts of cellular energy, are the most vulnerable to mitochondrial dysfunction [6]. Compared to age-matched controls, AD patients have significantly higher levels of mitochondrial DNA (mtDNA) deletions in large vulnerable neurons of the hippocampus and neocortex [7,8]. Interestingly, Down syndrome patients with AD dementia also have an increase in mtDNA mutations [9]. Aside from changes in mtDNA, AD mitochondria have decreased electron transport chain complex (ETC) IV, morphological changes in cristae, accumulation of osmiophilic material and decreased size [8,10].

There is evidence that Aβ-mediated toxicity may cause morphological, chemical and genetic changes in mitochondria. *In vitro* studies show that the accumulation of Aβ impairs mitochondrial biogenesis, dynamics and axonal transport [11-13]. Aβ also promotes oxidative stress, which can consequently introduce mutations in mtDNA and impair mitochondrial function [14-16]. In an *in vivo* study, severe toxicity was induced in the immediate vicinity of amyloid plaques, causing structural and functional abnormalities in mitochondria [17].

In our study, we sought to determine how Aβ mediated toxicity and Aβ pathology interacts with mitochondrial dysfunction *in vivo*. We modeled mitochondrial dysfunction *in vivo* by utilizing a mouse that contains a knockin mutation that inactivates the proofreading function of mitochondrial DNA polymerase-γ (*PolgA* D257A) [18]. The proofreading activity of mitochondrial DNA polymerase-γ has been shown both in mice and human cells to be critical for preventing accumulation of mtDNA mutations with age (reviewed in [19]). In humans, mutations in the *PolgA* gene cause various central nervous system disorders including cognitive decline [20]. In *PolgA* D257A mice, previous reports show that the animals rapidly develop a myriad of mitochondrial bioenergetic defects in multiple tissues, including the brain, and physical phenotypes that mimic premature ageing, including increased mortality after 1 year of age [18,21-23].

We crossed the *PolgA* D257A mice with a well-established transgenic AD mouse model carrying the APP familial London mutation (APPV717I; APP/Ld). APP/Ld mice do not exhibit neuron loss but develop amyloid plaques at ~1 year of age [24]. Since age is the greatest risk factor in Alzheimer’s disease and *PolgA* D257A mice exhibit a premature aging phenotype, we investigated whether *PolgA* D257A; APP/Ld bigenic mice may model the interaction between mitochondrial dysfunction associated with aging and Aβ toxicity in the onset and progression of AD. We hypothesized that mitochondrial dysfunction may affect the balance between Aβ synthesis and clearance, thus contributing to amyloidogenesis and potentially triggering neurodegeneration. Here, we provide evidence that the *PolgA* D257A mutation may increase β-amyloid accumulation by reducing IDE levels and thus impairing Aβ-clearance. In contrast, levels of the Aβ-generating enzymes BACE1 and PS1 or the APP C-terminal fragments (CTFs) C99 and C83 do not change in the presence of the *PolgA* D257A mutation. We also provide morphological and biochemical evidence of neurodegeneration in mice expressing the *PolgA* D257A mutation and APP/Ld transgene characterized by cortical and hippocampal atrophy and neuronal swelling and vacuolization. Our results suggest synergism between mitochondrial dysfunction and cerebral Aβ accumulation in some aspects of brain atrophy and neurodegeneration. These findings lend insights into the roles of mitochondrial dysfunction, amyloid pathology, and - more broadly - ageing in AD pathogenesis.

## Results

### Homozygous PolgA D257A mutation increases amyloid load in APP/Ld mice

Aging is the primary risk factor for AD, yet little is known regarding the mechanism of aging that drives AD pathogenesis. To model the effects that age-associated mitochondrial dysfunction may have on the development of amyloid pathology, we crossed *PolgA*^*D257A*/*D257A*^ mitochondrial DNA mutator mice (D257A mice; [18]) with transgenic mice that overexpress human APP harboring the London (V717I) familial AD (FAD) mutation (APP/Ld mice; [24]). We chose the APP/Ld mouse model because the transgenic APP sequence is wild-type at the BACE1 cleavage site, which allowed us to investigate the effects of mitochondrial mutations on BACE1 processing of APP in the absence of the BACE1-preferred APP Swedish mutation that may mask the effects of subtle changes in rates of BACE1 cleavage. This wild type APP BACE1 cleavage site more closely mimics BACE1 processing in sporadic AD. APP/Ld mice begin depositing amyloid at ~12 months of age [24]. Therefore, we aged monogenic and bigenic offspring of the above cross to ~12 months and analyzed the following 4 genotypes: 1) homozygous D257A; hemizygous APP/Ld, 2) homozygous D257A, 3) hemizygous APP/Ld, 4) wild-type.

**Figure 1:**
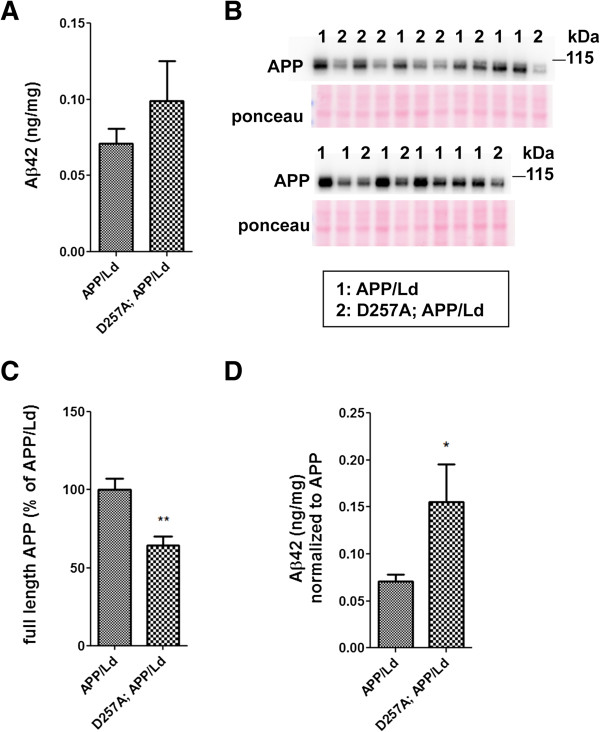
***PolgA *D257A mutation increases normalized Aβ42 levels in APP**/**Ld mice. (A)** Hemi-brain homogenates of ~12 month-old APP/Ld and D257A; APP/Ld mice were prepared and Aβ42 levels were measured using a human Aβ42-specific ELISA. Values are expressed in nanograms per milligram of total brain protein. Note that the raw Aβ42 levels are not significantly elevated in D257A; APP/Ld mice (n = 10), but that an upward trend exists, as compared to APP/Ld mice (n = 12). **(B)** 15 μg/lane of the same mouse brain homogenates in **(A)** were used for immunoblot analysis of full-length transgenic human APP/Ld protein using the anti-human APP monoclonal antibody 6E10. Ponceau S staining was used as a loading control. The numbers on immunoblot correspond to specific genotypes (as denoted in boxed legend key) of individual brain homogenates that were randomly loaded. **(C)** Full-length APP/Ld immunosignals in **(B)** were quantified by phosphorimager, normalized to Ponceau S staining intensities per lane, and expressed as percentage of the mean APP/Ld immunosignal. Note that levels of APP/Ld are significantly reduced in the D257A; APP/Ld mice compared to APP/Ld mice (mean ± SEM; **p < 0.01; Student’s t-test). **(D)** Raw Aβ42 ELISA values (ng/mg) from **(A)** were normalized to the mean transgenic APP/Ld protein levels for each genotype as determined in **(C)**. Note that Aβ42 levels normalized to APP/Ld are significantly increased in the D257A; APP/Ld mice compared to APP/Ld mice (mean ± SEM; *p < 0.05; Student’s t-test).

First, we determined whether the D257A mutation could increase cerebral levels of the toxic fibrillogenic 42-amino-acid isoform of Aβ (Aβ42) that accumulates in amyloid plaques and is associated with FAD. Whole brain homogenates from 12 month-old D257A; APP/Ld bigenic and APP/Ld mice were analyzed using a human Aβ42-specific ELISA (Figure 1A). Interestingly, D257A; APP/Ld mice exhibited a trend toward higher Aβ42 levels compared to age-matched APP/Ld littermate mice (p = 0.2585). One possible explanation of this upward Aβ42 trend could involve increased expression of the APP transgene in D257A; APP/Ld mice. To test this hypothesis, we performed immunoblot analysis of whole brain homogenates using the anti-human APP antibody 6E10 (Figure 1B,C). Unexpectedly, we observed that transgenic human APP levels in D257A; APP/Ld mice were only ~60% of those exhibited in APP/Ld mice. In contrast, endogenous mouse APP levels were unaffected by the presence of homozygous *PolgA*^*D257A*/*D257A*^ in D257A mice compared to wild-type littermates (Additional file 1: Figure S1A,B). The mechanism by which the D257A mutation reduces transgenic APP levels is presently unclear, although we speculate it may relate to a neurodegenerative process driven by a synergistic interaction between the effects of Aβ and those of the D257A mutation (see below). Most importantly, the D257A mutation resulted in a statistically significant ~2-fold increase in Aβ42 level when normalized to transgenic APP level in D257A; APP/Ld mice as compared to APP/Ld mice (Figure 1D). In other words, twice as much Aβ42 peptides per given amount of transgenic APP protein had accumulated in the brains of D257A; APP/Ld bigenic mice than in APP/Ld monogenic mice.

**Figure 2:**
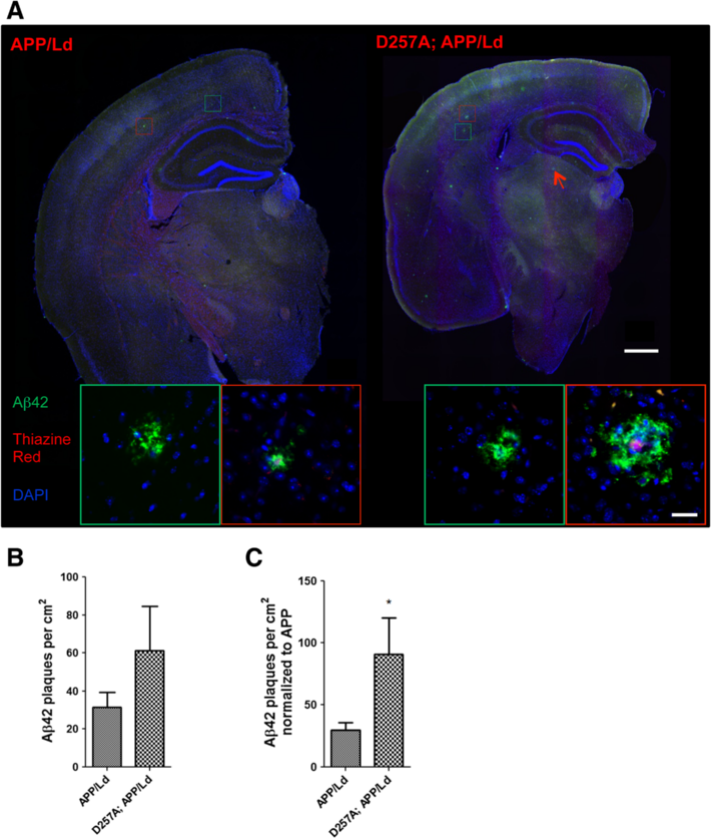
***PolgA *D257A mutation increases amyloid plaque pathology in APP**/**Ld mouse brains. (A)** Hemi-brains of the same mice from Figure 1 were fixed, sectioned coronally, and co-stained with anti-Aβ42 specific antibody (green) and thiazine red for β-sheet amyloid and imaged by immunofluorescence microscopy. **(A)** Representative coronal brain sections of APP/Ld (left) and D257A; APP/Ld mice (right). Both APP/Ld and D257A; APP/Ld mice exhibit diffuse (green box) and dense-core (red box) plaques. The insets show high-magnification images of plaques in green and red boxes. Dense-core plaques are positive for both Aβ42 and thiazine red, while diffuse plaques are only Aβ42-immunopositive. In addition to the cortex, D257A; APP/Ld mice form plaques in other brain regions such as the thalamus (for example see red arrow) Blue = DAPI. Scale bar: 500 μm (25 μm in insets). **(B)** The amyloid plaque density (number of Aβ42-positive plaques per cm^2^) was determined from representative brain sections with equivalent rostral-caudal location from D257A; APP/Ld (n = 10) and APP/Ld (n = 12) mice. **(C)** Plaque densities in **(B)** were normalized to human transgenic APP protein levels as determined by APP immunoblot analysis (Figure 1C). Note that normalized Aβ42 plaque density is significantly increased in D257A; APP/Ld mice compared to APP/Ld mice (mean ± SEM; *p < 0.05; Student’s t-test).

Next, we determined whether the ~2-fold elevation in normalized Aβ42 level caused by the D257A mutation translated into increased amyloid plaque number in D257A; APP/Ld transgenic mice. Coronal brain sections from the same 12 month-old mice as above were co-stained with thiazine red (for β-sheet amyloid) and anti-Aβ42-selective antibody, imaged by fluorescence microscopy and counted for Aβ42-positive plaques (Figure 2). Diffuse and dense-core plaques were relatively small in size and sparsely distributed throughout the cortex and hippocampus of D257A; APP/Ld and APP/Ld mice (Figure 2A), as expected for this relatively early stage of plaque formation in this AD mouse model. Interestingly, plaques were observed in the thalamus of D257A; APP/Ld mice, but were largely absent in the thalamus of APP/Ld mice. D257A; APP/Ld mice exhibited a trend toward higher amyloid plaque density, expressed as Aβ42-positive plaques per total area analyzed, compared to age-matched APP/Ld littermate mice (p = 0.2109) (Figure 2B). As with normalized Aβ42 levels by ELISA (Figure 1D), the plaque densities normalized to transgenic APP levels were significantly increased by over 2-fold in D257A; APP/Ld mice compared to APP/Ld mice (p < 0.05) (Figure 2C). Moreover, amyloid plaque size appeared to be larger on average in D257A; APP/Ld brains relative to APP/Ld (Figure 2A right, insets outlined in red). Thus, homozygous D257A mutation led to greater amyloid plaque loads in the brains of APP/Ld transgenic mice.

### Homozygous PolgA D257A mutation prevents APP/Ld transgene-induced IDE elevation but does not affect levels of NEP or Aβ generating enzymes in APP/Ld mice

**Figure 3:**
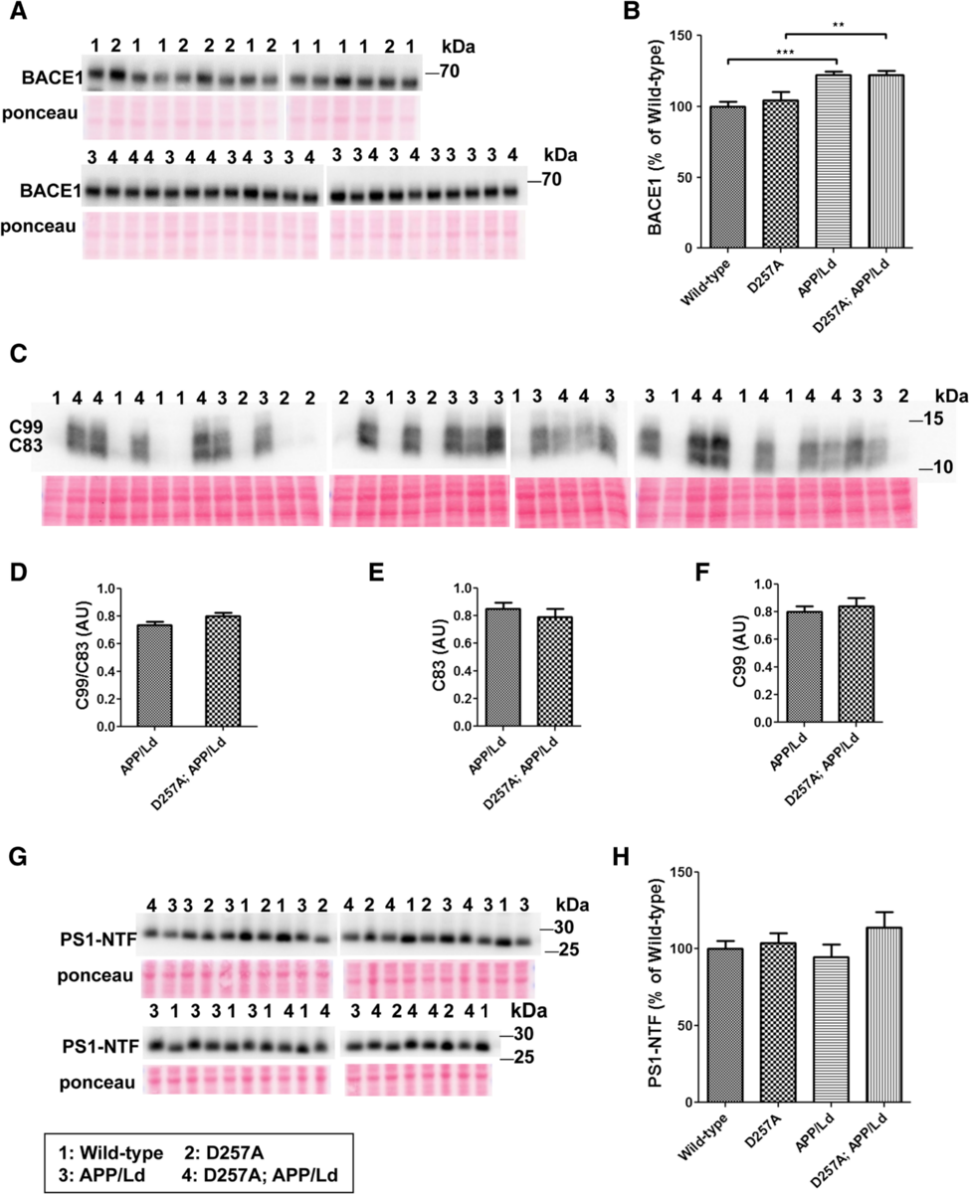
***PolgA *D257A mutation does not affect levels of Aβ generating enzymes in APP/Ld mice.** 15 μg of brain homogenates from D257A; APP/Ld, D257A, APP/Ld, and wild type mice were loaded per lane and subjected to immunoblot analysis. Immunoblot signals were normalized to the Ponceau S staining intensity for a given lane. The numbers on the immunoblot correspond to mice with specific genotypes as denoted in the legend key (box). **(A)** BACE1 protein runs at ~68 kDa. **(B)** BACE1 immunosignals in **(A)** were expressed as the percentage of the mean wild-type mouse BACE1 immunosignal. Note that BACE1 levels are significantly higher in APP/Ld monogenic and D257A; APP/Ld bigenic mice compared to D257A and wild type non-transgenic mice. BACE1 levels are not altered by the presence of the D257A mutation (mean ± SEM; **p < 0.01; ***p < 0.001; One-way ANOVA followed by Newman–Keuls multiple-comparisons *post hoc* test). **(C)** APP processing was determined by measuring levels of both APP C-terminal fragments (C83 & C99) via immunoblot using a rabbit monoclonal antibody that recognizes the C-terminus of APP. Note however, that these C-terminal fragments were below the level of immunoblot detection in non-transgenic APP mouse brains. **(D)** The ratio of C99:C83, which is directly proportional to the fraction of APP cleaved by BACE1, is not altered by the presence of the D257A mutation. Separate quantification of C83 **(E)** and C99 **(F)**, normalized to Ponceau S, also show that α- and β-secretase activities are not altered independently of one another by the D257A mutation. **(G)** PS1 protein levels were measured by immunoblot analysis using a PS1 N-terminal antibody. **(H)** PS1 immunosignals in **(G)** were expressed as the percentage of the mean wild-type control PS1 level. PS1-NTF levels are not altered in the presence of the D257A mutation or the APP/Ld transgene.

In principle, the elevation of normalized amyloid load in D257A; APP/Ld mice could have resulted from either an increase in Aβ production or a decrease in Aβ clearance and/or degradation. To address the question of increased Aβ production first, we measured the levels of Aβ generating enzymes BACE1 and presenilin 1 (PS1) by immunoblot analysis on whole brain homogenates of 12 month-old D257A; APP/Ld, APP/Ld, D257A, and wild-type mice (Figure 3). BACE1 is the key enzyme that initiates the production of Aβ [25-31]. Consistent with our previous analyses of other APP transgenic mice [32-34], mice expressing the human APP/Ld transgene exhibited increased cerebral levels of BACE1 protein compared to wild-type non-transgenic control mice (Figure 3A,B). D257A; APP/Ld bigenic mice also displayed increased BACE1 levels, but homozygous D257A mutation did not enhance the APP transgene-induced BACE1 elevation. Mice harboring the D257A mutation alone showed similar BACE1 levels as wild-type controls. To investigate whether the D257A mutation may have enhanced BACE1 activity independent of BACE1 level, we analyzed the levels of the BACE1-cleaved C99 APP CTF and the α-secretase processed CTF, C83 (Figure 3C). Since BACE1 and α-secretase compete to cleave APP under some circumstances [25], an increase in the C99:C83 ratio may indicate a shift towards higher BACE1 activity. However, we observed no difference in the C99:C83 ratio between D257A; APP/Ld and APP/Ld mice (Figure 3D). Additionally, the individual levels of C83 and C99 did not change in D257A; APP/Ld mice as compared to APP/Ld mice, suggesting that the D257A mutation does not alter activities of either α- or β-secretase independently (Figure 3E,F). Finally, we examined whether PS1/γ-secretase levels were changed in the presence of the D257A mutation by performing immunoblot analysis for PS1 N-terminal fragment (NTF) (Figure 3G,H). Presenilin is the catalytic subunit of the γ-secretase complex that, in addition to BACE1, is also required for Aβ generation. No changes in PS1 NTF levels were observed in the brains of mice regardless of genotype. Taken together, these findings indicate that homozygous D257A mutation did not significantly affect the protein and activity levels of Aβ generating enzymes in the brains of transgenic mice, suggesting that that elevation of the normalized Aβ42 level and amyloid plaque load observed in D257A; APP/Ld mice was unlikely the result of increased Aβ production.

**Figure 4:**
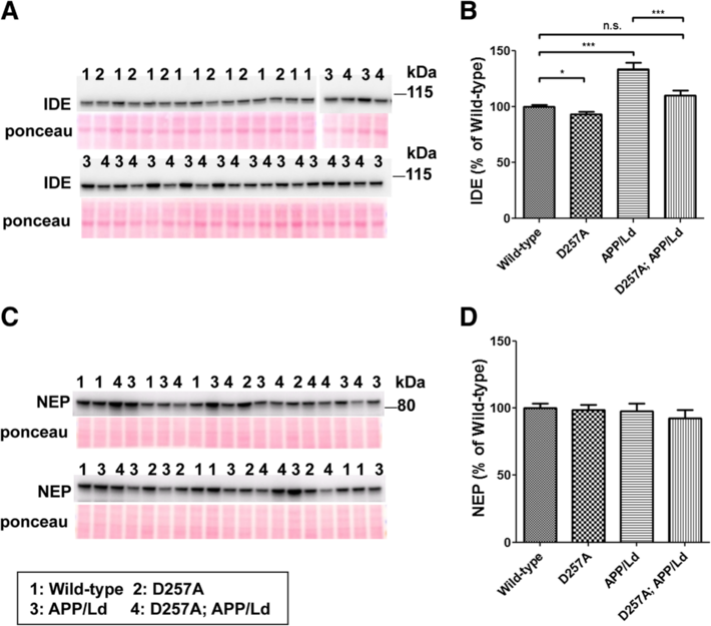
***PolgA *D257A mutation prevents Aβ**-**induced IDE elevation.** Brain homogenates were prepared from D257A; APP/Ld, D257A, APP/Ld, and wild type mice and 10 μg protein/lane was randomly loaded and subjected to immunoblot analysis for two Aβ degrading enzymes: insulin degrading enzyme (IDE) and neprilysin (NEP). Immunoblot signals were normalized to Ponceau S staining intensities in a given lane. The numbers on immunoblot correspond to a mouse with a specific genotype as denoted in the legend key (box). **(A)** IDE protein from brain homogenates runs at ~100 kDa. **(B)** IDE immunosignals from **(A)** were measured and expressed as percentage of mean wild-type IDE level. Note that IDE levels are reduced slightly in the brains of D257A mice. As previously reported for APP transgenic mice [38,39], IDE levels are significantly increased in the brains of APP/Ld mice compared to wild-type non-transgenic mice. Most importantly, the D257A mutation completely prevents the Aβ-induced increase in IDE levels in D257A; APP/Ld mice (mean ± SEM; n.s. = not significant; *p < 0.05; ***p < 0.001; One-way ANOVA followed by Newman–Keuls multiple-comparisons *post hoc* test). **(C)** NEP protein from brain homogenates runs at ~90 kDa. **(D)** NEP immunosignals from **(C)** were measured and expressed as percentage of mean wild-type NEP level. Note that NEP levels are not altered by the D257A mutation, the APP/Ld transgene, or their combination, as compared to wild-type non-transgenic mice.

Next, we explored the possibility that cerebral amyloid accumulation in D257A; APP/Ld mice could be due to impaired Aβ degradation. Insulin degrading enzyme (IDE) and neprilysin (NEP) are major Aβ degrading enzymes in the brain [35-37]. It has been previously reported that IDE levels become elevated in response to Aβ [38,39], suggesting a feedback mechanism of IDE regulation for lowering cerebral Aβ levels. We performed immunoblot analysis for IDE and NEP levels in whole brain homogenates from 12 month-old D257A; APP/Ld, APP/Ld, D257A, and wild-type mice (Figure 4). Consistent with previous reports [38,39], IDE levels were significantly elevated in response to Aβ accumulation in the brains of APP/Ld compared to wild-type mice (Figure 4A,B). We also observed a small but significant decrease of IDE level in D257A mice relative to wild-type. Importantly, the Aβ-induced IDE increase was completely abrogated by the presence of homozygous D257A mutation in D257A; APP/Ld bigenic mice. In contrast, NEP levels were unchanged regardless of genotype in the brains of transgenic mice (Figure 4C,D). Together, these results suggest the intriguing possibility that the elevated Aβ42 level and amyloid plaque load in D257A; APP/Ld bigenic mice is the result of D257A-associated inhibition of the Aβ-induced IDE increase, thus leading to reduced IDE-mediated degradation of Aβ.

### Homozygous PolgA D257A mutation and APP/Ld transgene expression synergize to cause brain atrophy and neurodegeneration

**Figure 5:**
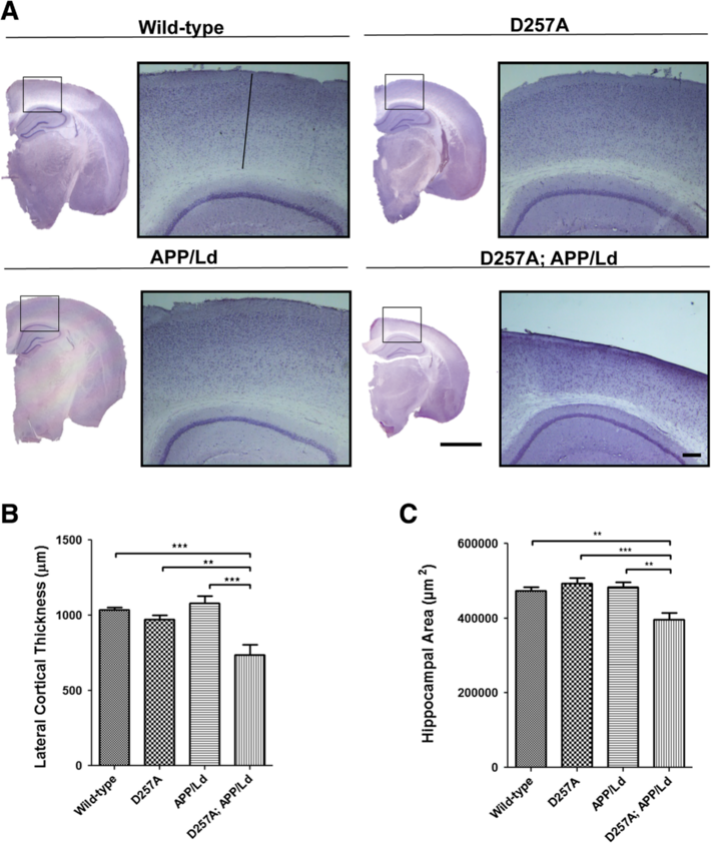
**Co**-**expression of the *PolgA *D257A mutation and APP**/**Ld transgene causes brain atrophy. (A)** Coronal sections (30 μm) of brains from D257A; APP/Ld, D257A, APP/Ld, and wild type mice were selected from similar rostral-caudal positions, stained with hematoxylin and imaged by light microscopy. Mouse genotypes are indicated above the micrographs. High magnification images of cortical regions dorsal to the hippocampus (boxed areas) are presented to the right of each low magnification image. Note that the brains of D257A; APP/Ld mice are significantly smaller in size compared to the other genotypes. Especially noticeable is the reduced cortical thickness of D257A; APP/Ld mice. (scale bar = 250 μm for high magnification, 2 mm for low magnification). **(B)** Lateral cortical thickness (μm) was measured as the length of a line (drawn in the wild-type high magnification image in **(A)**) running perpendicular to the cortical layers from the dorsal edge of the CA1 region of the hippocampus to the visible boundary of the cortex. The brains of D257A; APP/Ld mice show significant thinning of the cortex compared to the other genotypes (n = 5; mean ± SEM; *p < 0.05; One-way ANOVA followed by Newman–Keuls multiple-comparisons *post hoc* test). **(C)** Hippocampal area (μm^2^) of the different genotypes was also measured. Note that D257A; APP/Ld mice also exhibit significantly reduced hippocampal area compared to APP/Ld, D257A or wild-type mice (n = 5; mean ± SEM; **p < 0.01; ***p < 0.001; One-way ANOVA followed by Newman–Keuls multiple-comparisons *post hoc* test).

Unlike in AD patients, the brains of most lines of APP transgenic mice do not exhibit significant brain atrophy. However, during the course of our study, we noticed that the brains of 12 month-old D257A; APP/Ld mice appeared smaller compared to brains of the other genotypes, suggesting brain atrophy. To investigate this further, we performed hematoxylin staining on coronal brain sections and found that significant shrinkage of the brain had occurred only in the combined presence of the homozygous D257A mutation and the APP/Ld transgene (Figure 5A). Analysis of the cortex and hippocampus revealed that D257A; APP/Ld mice exhibited ~70% and ~80% of the lateral cortical thickness and hippocampal area of mice of the other genotypes, respectively (Figure 5B,C). These results demonstrate the presence of significant brain atrophy only in the D257A; APP/Ld mice, suggesting synergism between the effects of homozygous *PolgA* D257A mutation and APP/Ld transgene expression leading to reduced brain size.

**Figure 6:**
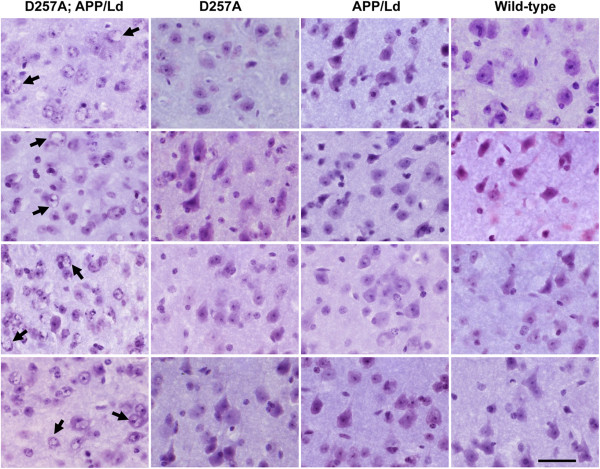
**Co**-**expression of the *PolgA *D257A mutation and APP**/**Ld transgene causes morphological features of neurodegeneration.** Coronal sections (30 μm) of brains from D257A; APP/Ld, D257A, APP/Ld, and wild type mice were selected from similar rostral-caudal positions, stained with hematoxylin, and cortical layer V neurons imaged at high magnification by light microscopy. Mouse genotypes are indicated above each column. Representative brain sections from four different mice from each genotype were imaged at 100× magnification. Note that D257A; APP/Ld neurons have abnormal morphologies and display vacuolated nuclei suggestive of marginated chromatin (e.g., black arrows). These features are absent in neurons in D257A, APP/Ld and wild-type brains (scale bar: 50 μm).

While examining hematoxylin stained sections, we observed that many cortical and hippocampal neurons of D257A; APP/Ld bigenic mice exhibited morphological features suggestive of apoptosis, including clear cytoplasm and swollen deformed vacuolated nuclei with marginated chromatin (Figure 6). These neuronal phenotypes were very rare or absent in neurons of APP/Ld, D257A, or wild-type mice. Interestingly, a variety of histopathological studies have documented similar morphological features of apoptosis in neurons of AD brains (reviewed in [40]).

**Figure 7:**
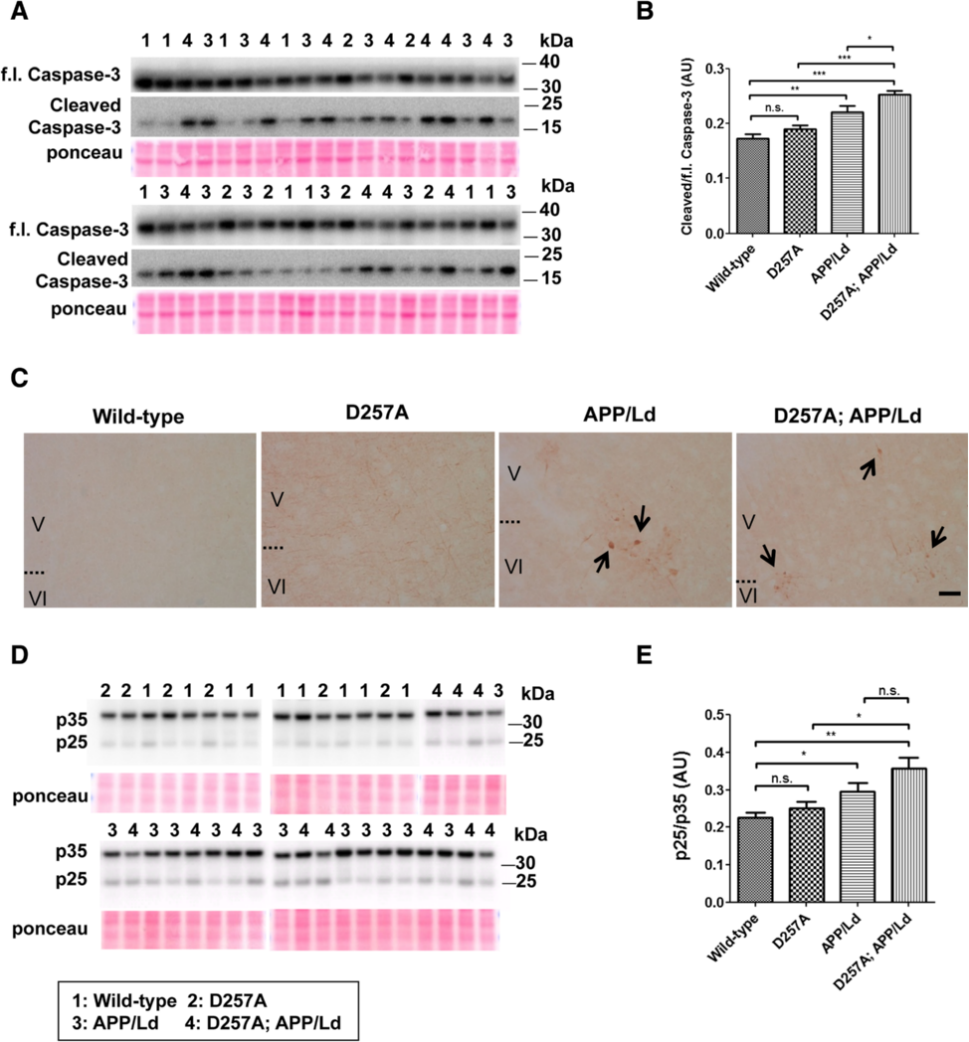
**Co-expression of the *PolgA *D257A mutation and APP/Ld transgene causes increased levels of neurodegeneration markers, cleaved caspase-3 and p25.** 30 μg of brain homogenates from D257A; APP/Ld, D257A, APP/Ld, and wild type mice were randomly loaded and subjected to immunoblot analysis for cleaved caspase-3 fragment and p25. Immunoblot signals were normalized to Ponceau S staining intensities in a given lane. The numbers on immunoblot correspond to a mouse with a specific genotype as denoted in the legend key (box). **(A)** Full-length (f.l.) and activated cleaved caspase-3 run at ~35 kDa and ~17 kDa respectively, as previously reported [33]. **(B)** Ratio of immunosignal intensities of cleaved to f.l. caspase-3. Note that the cleaved:f.l. caspase-3 ratio is significantly increased in APP/Ld mice, although D257A; APP/Ld mice have an even greater cleaved:f.l. caspase-3 ratio, suggesting synergism between the D257A mutation and APP/Ld transgene. **(C)** DAB staining using cleaved caspase-3 neoepitope antibody on coronal sections (30 μm) demonstrate cleaved caspase-3-positive puncta in cell bodies of cortical neurons (cortical layers V and VI) of APP/Ld and D257A; APP/Ld mice (black arrows; scale bar: 100 μm). No immunoreactivity for cleaved caspase-3 is observed in wild-type and D257A mice. **(D)** Immunoblots using an antibody that recognizes both p35 and the neurodegeneration marker, calpain-cleaved p25 fragment. **(E)** Ratio of immunosignal intensities of p25 to p35. Note that the p25:p35 ratio is significantly elevated in APP/Ld and D257A; APP/Ld mice compared to wild-type mice. Increased p25:p35 ratio is seen in D257A; APP/Ld compared to APP/Ld mice suggesting synergism, although this trend did not reach statistical significance. D257A mice do not have an elevation of p25:p35 ratio (mean ± SEM; n.s. = not significant; *p < 0.05; **p < 0.01; One-way ANOVA followed by Newman–Keuls multiple-comparisons *post hoc* test).

Caspase activation, a biochemical marker of apoptosis, has been reported in AD [41,42] and in different lines of APP transgenic mice [43-46]. To investigate whether apoptosis might be associated with the brain atrophy observed in D257A; APP/Ld mice, we measured levels of the cleaved 17 kDa fragment of caspase-3 as a marker of capase-3 activation. Immunoblot analysis of whole brain homogenates with an antibody that recognizes the neoepitope of 17 kDa cleaved caspase-3 showed that cleaved caspase-3 levels were nearly 50% higher in the brains of 12 month D257A; APP/Ld mice than in wild-type or D257A mice (Figure 7A,B). As expected, cleaved caspase-3 levels were also elevated in brains of APP/Ld mice, but to a lesser extent than in D257A; APP/Ld mice. In addition, we immunostained coronal brain sections with the cleaved caspase-3 neoepitope antibody and observed cleaved caspase-3 positive puncta in the cortex of D257A; APP/Ld and APP/Ld mice, but not in wild-type or D257A mice (Figure 7C). These caspase-3 positive puncta appeared similar in size, shape and distribution as those reported in the brains of other APP transgenic mice [43,46]. Together, these results suggest that the D257A mutation exacerbates APP/Ld transgene-associated activation of caspase-3 in neurons of the brain, thus promoting apoptosis.

The calpain-cleaved fragment of the p35 regulator of Cdk5, termed p25, is associated with neurodegeneration in humans and animal models (reviewed in [47]). To determine whether p25 levels were elevated in the presence of the D257A mutation, we performed immunoblot analysis of whole brain homogenates with an antibody that recognizes both p35 and p25 (Figure 7D,E). Similar in magnitude to the increase in cleaved caspase-3 level, the p25:p35 ratio was ~50% higher in the brains of 12 month-old D257A; APP/Ld than in wild-type or D257A mice (Figure 7E). The p25:p35 ratio in APP/Ld mice was significantly increased compared to wild-type mice, as observed in other APP mice [33,34,48]. The increased p25:p35 ratio observed in D257A; APP/Ld mice compared to APP/Ld did not reach statistical significance but further corroborated the observed neurodegeneration in D257A; APP/Ld mice.

**Figure 8:**
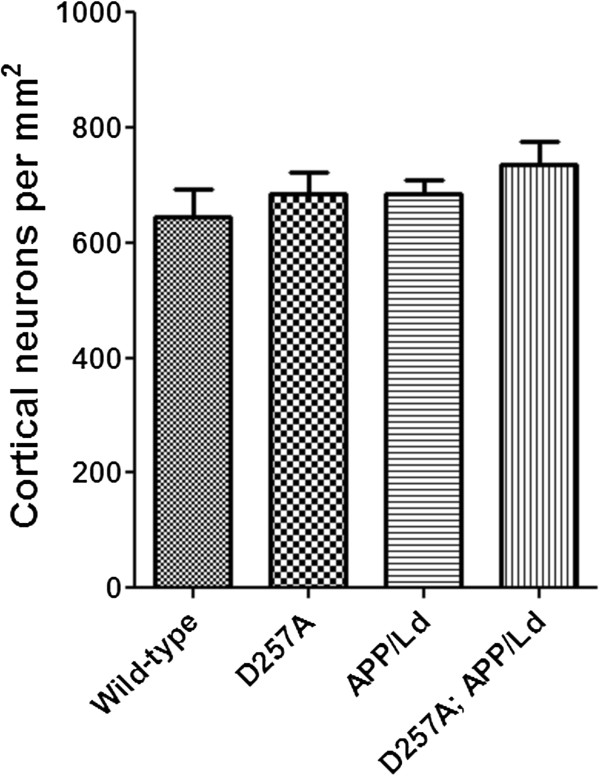
**Co**-**expression of the *PolgA *D257A mutation and APP/Ld transgene does not cause frank neuron loss.** Representative coronal sections (30 μm) of brains from D257A; APP/Ld, D257A, APP/Ld, and wild type mice were selected from similar rostral-caudal positions, stained with hematoxylin, and images centering on cortical layer V were obtained at 100× magnification by light microscopy. Neuron density (number of neurons per mm^2^) was determined by counting the number of neurons in representative 100× fields of middle layers of dorsal cortex for the four genotypes of mice. Note that no significant differences in neuron densities were observed between the different genotypes (n = 5 mice/genotype; mean ± SEM; One-way ANOVA test followed by Newman–Keuls multiple-comparisons *post hoc* test).

The brain atrophy, vacuolated neurons, caspase-3 activation, and increased p25 levels in D257A; APP/Ld mice suggested that the bigenics might have neuron loss compared to the other genotypes. To investigate this possibility, we performed neuron counting in middle layers of dorsal cortex in hematoxylin stained coronal brain sections from the same relative rostral-caudal positions for the four genotypes of mice (Figure 8). Neuron density was determined in regions that included vacuolated neurons and significant cortical atrophy in the D257A; APP/Ld mice (Figure 5A,B). Remarkably, we did not observe significant differences in neuron densities between any of the genotypes. In retrospect, perhaps this is not surprising, given that few APP transgenic mouse models exhibit significant neuron death, while most do show signs of neurodegeneration such as synaptic loss, caspase-3 activation, and increased p25. Taken together, our results suggest that synergism between the *Polg A* D257A mutation and the APP/Ld transgene in D257A; APP/Ld mice results in enhanced neurodegeneration in the absence of frank neuron loss.

## Discussion

Age is the primary risk factor for AD, yet the mechanism responsible for this association remains enigmatic. Reduced mitochondrial function is hypothesized to occur during aging and in AD (reviewed in [5]) and may result from age-associated accumulation of point mutations and deletions in mtDNA (reviewed in [49]). Neuronal populations are potentially more vulnerable to mitochondrial dysfunction due to their high bioenergetic demands, thus providing a possible mechanistic link between aging, mitochondrial dysfunction and AD.

Despite strong evidence that cerebral Aβ accumulation plays an early part in AD pathogenesis, the precise mechanism of Aβ neurotoxicity remains elusive. Several APP transgenic mouse models of AD successfully recapitulate key features of the disease such as amyloid plaque pathology, but fail in other aspects such as neurodegeneration (reviewed in [50]). Interestingly, intracerebral injection of fibrillar Aβ in aged but not young primates resulted in neuronal loss, suggesting that Aβ neurotoxicity is a pathological response of the aging brain [51]. In our study, we employed mitochondrial DNA mutator *PolgA* D257A mice that accumulate mtDNA mutations with age to test the hypothesis that age-related mitochondrial dysfunction can exacerbate amyloid pathology and induce neurodegeneration in APP transgenic mice.

A major finding in our study was that both Aβ42 levels and amyloid plaque load were increased in the brains of D257A; APP/Ld mice when normalized to transgenic APP/Ld protein levels. Therefore, the D257A mutation exacerbated the cerebral accumulation of Aβ42 per given amount of APP in the bigenic mice. Although trends toward increased absolute Aβ42 levels and amyloid plaque density were observed in the bigenic mice, they became statistically significant when normalized to transgenic APP/Ld levels (Figures 1 and 2). Our immunoblot analysis for human APP revealed that there was close to 40% reduction in APP/Ld protein levels in D257A; APP/Ld bigenic mice compared to the parental APP/Ld monogenic mice. In contrast, endogenous mouse APP levels were unaffected in D257A monogenic mice, demonstrating that the *PolgA* mutation alone does not alter APP expression levels. We speculate that the reduced levels of APP/Ld protein in brains of bigenic mice might relate to neurodegenerative processes that were occurring prior to neuron death. Bigenic neurons appeared to be in a severe state of degeneration (Figure 6) and showed caspase-3 activation and increased p25 levels (Figure 7). Given their state of pathology, degenerating D257A; APP/Ld neurons subject to Aβ neurotoxicity could well have reduced APP/Ld transgene transcription or translation. Alternatively, APP/Ld in D257A; APP/Ld mice could have undergone cleavage by increased levels of activated caspase-3 [52], thus lowering total APP/Ld levels. Future studies will be necessary to clarify the mechanism of decreased APP/Ld levels in D257A; APP/Ld bigenic brains.

The exacerbated cerebral accumulation of Aβ in D257A; APP/Ld mice could have resulted from either increased production or decreased clearance/degradation of Aβ. Because we did not observe any changes in the levels of BACE1, PS1, C99, and C83 in D257A; APP/Ld bigenic versus APP/Ld mice (Figure 3), all evidence supporting increased Aβ production in the brains of the bigenic mice is lacking. It should be noted that BACE1 levels were significantly increased in the brains of D257A; APP/Ld and APP/Ld compared to D257A and wild-type mice, consistent with our previous work showing that BACE1 levels are elevated by amyloid pathology in human AD and APP transgenic mouse brains [32,33,53]. Nevertheless, the lack of support for a D257A-associated increase in Aβ production suggested that impaired Aβ clearance/degradation might have led to the increase of Aβ accumulation in D257A; APP/Ld brain. To test this hypothesis, we analyzed cerebral levels of two major Aβ degrading enzymes, IDE and NEP. Consistent with findings from other APP transgenic mice [38,39], levels of IDE, but not NEP, were increased in response to Aβ accumulation in the brains of APP/Ld mice (Figure 4). This Aβ-induced increased IDE level might reflect a feed-back mechanism that attempts to counteract the build-up of Aβ in the brain. Additionally, IDE strongly prefers to localize within GFAP-positive astrocytes surrounding Aβ plaques [39]. Most importantly, in the D257A; APP/Ld bigenic mice, we observed that the D257A mutation completely abrogated the Aβ-induced increase in IDE level. Our study is the first to show *in vivo* that mitochondrial dysfunction, by the D257A mutation, can prevent the Aβ-induced IDE increase. Interestingly, a previous report indicated that the D257A mutation can affect metabolism by changing levels of major hormones such as leptin and ghrelin [54]. Therefore, it is not unprecedented that the D257A mutation can affect enzymes in metabolic pathways, like IDE. Additionally, D257A; APP/Ld mice phenotypically mimic aspects of advanced aging and increased mortality at time of analysis at 12 months of age. Thus, the abrogated IDE increase in these mice might represent an aging-related effect [55]. Because Aβ peptide levels have been shown to correlate inversely with IDE levels *in vivo*[37,56], the blocked Aβ-induced IDE increase could be responsible for the exacerbated amyloid accumulation in the bigenic mice.

Previous studies of APP/Ld transgenic mice show absence of substantial brain atrophy [24,57], implying that Aβ accumulation on its own does not cause significant neurodegeneration. Indeed, our study supports a two-hit hypothesis whereby Aβ is insufficient to cause substantial neurodegeneration by amyloid buildup alone. An additional stress factor, such as mitochondrial dysfunction, is proposed to combine with Aβ to trigger the anticipated cascade of events leading to neurodegeneration.

A growing body of work from two independent knockin mouse lines has demonstrated that the *PolgA* D257A mutation causes increased accumulation of mtDNA mutations that leads to progressive mitochondrial dysfunction with age [18,21,22,58-63]. Our work suggests the possibility that Aβ toxicity might exacerbate mitochondrial dysfunction associated with the D257A mutation, thus causing brain atrophy. Future studies should be undertaken to investigate whether Aβ can worsen pre-existing age-related mitochondrial deficits and their effects on neurodegeneration.

Hematoxylin staining of coronal brain sections revealed that only the bigenic D257A; APP/Ld mice exhibited a smaller overall brain size with shrinkage of the cortex and hippocampus, compared to the other genotypes (Figure 5). Additionally, cortical neurons in the bigenic mice revealed abnormal nuclear morphologies reminiscent of neurons undergoing apoptosis [40,64]. In contrast, cortical neurons of D257A and APP/Ld mice appeared morphologically similar to those of wild-type mice. A time-course study examining morphological changes in the brains of D257A; APP/Ld compared to the other genotypes will be important for determining when these abnormalities appear.

We also observed that the D257A; APP/Ld mice bigenic mice exhibited significantly increased levels of activated caspase-3 17 kDa fragment compared to the other genotypes (Figure 7). Activated caspase-3 levels were also increased in APP/Ld mice, but were less elevated compared to the bigenic mice. We corroborated our biochemical analysis with immunohistochemical staining of brain sections that revealed activated caspase-3-positive puncta in layer V/VI cortex in D257A; APP/Ld and APP/Ld mouse brains. Interestingly, in D257A mice post-mitotic tissues such as the brain have been shown to be more resistant to the induction of apoptosis by mtDNA mutations [18]. However, the bigenic mice carrying the D257A mutation appeared to have reduced resistance to apoptosis. These results are significant because activated caspase-3 immunoreactivity has been observed in AD brain [42] and in other APP transgenic mouse models [43,65]. Although the underlying mechanism of brain atrophy in the D257A; APP/Ld mice is not known, it might involve Aβ42-induced mitochondrial cytochrome c release and subsequent activation of caspase-3 to trigger apoptosis [66]. Importantly, significant activation of caspase-3 can lead to eventual neuron loss (reviewed in [40,67,68]). Additionally, the levels of p25, a marker of neurodegeneration, were the highest in bigenic mice compared to the other genotypes (Figure 7).

Although we did not observe frank neuron loss in D257A; APP/Ld mice by cell counting (Figure 8), we suspect that neurons were in the process of dying, given the brain atrophy, vacuolated neurons, activated caspase-3, and elevated p25 levels of the bigenic mice. *PolgA* D257A knockin mice do not live long past one year [18], and we observed a dramatic increase in mortality of D257A; APP/Ld mice at that age. Therefore, we speculate that we would have observed frank neuron loss in the bigenic mice had they been able to live longer. We hypothesize that the observed brain atrophy and increased markers of neurodegeneration are primarily the result of axon and dendrite degeneration, synaptic loss, white matter loss, or a combination of these factors, which precede neuron death.

Finally, oligomeric forms of Aβ are widely thought to underlie important aspects of synaptic dysfunction and neurodegeneration in AD (reviewed in [69]). Although we did not examine Aβ oligomers in the current study, the possibility exists that the D257A mutation might affect the proportion or toxicity of Aβ oligomers in the brains of D257A; APP/Ld bigenic mice. It will be important to conduct future studies to address the potential influence of the D257A mutation on Aβ oligomer level, form, and neurotoxicity.

## Conclusions

Taken together, the cortical thinning, reduction of hippocampal area, aberrant neuronal morphology, and increased activated caspase-3 and p25 all suggest that neurodegeneration in the absence of frank neuron loss is likely to be the underlying cause of brain atrophy of the D257A; APP/Ld mice. Moreover, this neurodegenerative phenotype appears to have resulted from synergism between Aβ neurotoxicity and mitochondrial dysfunction leading to reduced Aβ-associated IDE induction, increased Aβ accumulation, and ultimately brain atrophy. Our results support a role for age-dependent mitochondrial dysfunction in AD pathogenesis.

## Methods

### Mice

APPV717I (APP/Ld) mice were generated and characterized previously [24]. These mice were crossbred to the *PolgA*^*D257A*/*D257A*^ mitochondrial DNA mutator mice [18]. At 12 months of age, the mice were deeply anesthetized with ketamine/xylazine and transcardially perfused with PBS containing protease (Cocktail Set III, Animal-free, Cat#: 535140, Calbiochem) and phosphatase (Halt Phosphatase Cocktail Prod#: 1861277 Thermo Scientific) inhibitors. Brains were excised and one hemibrain was drop fixed in 4% PFA and cryopreserved in 30% sucrose, PBS for immunohistochemistry, while the other hemibrain was flash frozen in liquid nitrogen for biochemical analysis. All mice were maintained in microisolator cages in the Barrier Facilities of Northwestern University Center for Comparative Medicine. All animal procedures were in strict accordance with the National Institutes of Health Guide for the Care and Use of Laboratory Animals and were Northwestern University Animal Care and Use Committee approved.

### Tissue preparation for biochemical analysis

Hemibrains were flash frozen in liquid N2 and stored at -80°C. Frozen mouse hemibrains were homogenized in 1× PBS, 1% Triton X-100, 1× protease inhibitor cocktail (Calbiochem), and 1× Halt phosphatase inhibitor cocktail (Thermo Scientific). Total protein concentration was determined by the BCA method (Pierce).

### Human Aβ42 ELISA

Total Aβ42 levels in brain homogenates were determined using a human Aβ (1-42) ELISA kit (Wako Pure Chemical Industries, Ltd.), according to manufacturer’s recommendations. Briefly, APP/Ld and D257A; APP/Ld brain homogenates (~1 mg total protein) was extracted for four hours in 5 M guanidine-HCl at room temperature (300 μl total volume). Homogenates were further diluted 10-fold in ice-cold casein buffer (0.25% casein/0.05% sodium azide/5 mmol/l EDTA, pH 8.0 in PBS with 1× protease inhibitor cocktail (Calbiochem), centrifuged at 16,000 g for 20 min at 4°C, and then diluted again 1:50 with Standard diluent. Samples were run in duplicates on Aβ42-specific ELISA (100 μl/well). Optical densities (450 nm) of each well were read on a Spectra Max 250 plate reader (Molecular Devices Corp., Sunnyvale, CA), and sample Aβ42 concentrations were determined by comparison with the Aβ42 standard curves. Each reading was conducted in the linear range of the assay. Aβ42 concentration values were normalized to total brain protein concentrations and were expressed as nanograms of Aβ42 per milligram total protein, and the average of the duplicates was defined as the Aβ42 concentration for a given mouse.

In order to normalize Aβ42 concentration to APP transgenic protein level for each mouse, the Aβ42 concentration was divided by a normalization factor (NF) calculated for a given mouse. To do so, immunoblots were performed on mouse brain homogenates to measure full-length human APP levels using the 6E10 antibody (see Immunoblotting Section). Following enhanced chemiluminescence detection, the raw sum intensities of APP immunoblot band signals for all mice were determined and normalized to Ponceau S staining. Next, the mean of the normalized APP immunoblot signal intensities for the APP/Ld mice as a group was determined. The APP immunoblot signal intensity for each APP/Ld and D257A; APP/Ld mouse was then divided by the APP/Ld mean intensity to derive an individual NF for every mouse. Finally, a given Aβ42 ng/mg total protein ELISA value was divided by the individual NF for each mouse to calculate the mouse’s Aβ42 concentration normalized to APP transgenic protein level, and then means and SEMs for each genotype were determined.

### Immunoblotting

In general, 10-30 μg protein from whole-brain homogenates were heated at 95°C in sample boiling buffer (60 mM Tris HCl, 5% SDS, 10% glycerol, pH = 6.8) with 5× loading dye (90% BME, 10% of 5% Bromophenol blue dye) before SDS-PAGE separation on 4%–12% NuPAGE Bis-Tris gels in 1× MES or MOPS running buffer (Invitrogen, Carlsbad, CA). Alternatively, samples for PS1-NTF immunoblot were not boiled but were otherwise prepared similarly. For C99 and C83 immunoblots, samples were separated on 16% Tris-glycine gels using 1× Tris-glycine as a cathode buffer and 200 mM Tris-base (pH 8.8) as an anode buffer. Proteins were electrophoretically transferred onto Millipore Immobilon-P polyvinylidene difluoride (PVDF) membrane (Millipore, Billerica, MA). Multiple protein gels were run in parallel to accommodate the large number of samples. When conducting the protein transfer step, gels were aligned horizontally and transferred onto a single piece of PVDF membrane. This process ensured consistency as all samples then were developed on the same PVDF membrane. After completing protein transfer, PVDF membranes were stained with Ponceau S and imaged on a scanner. Blots were then blocked in 5% non-fat dry milk in Tris-buffered saline (TBS), 0.1% Tween 20 (TBST; Sigma) for 1 h at room temperature, then incubated in primary antibody (human APP: Aβ Mouse mAb 6E10, Chemicon Cat. # MAB1560, 1:1,000; human and mouse APP: Anti-Alzheimer Precursor Protein A4 Mouse mAb N-term specific, 22C11, Millipore Cat. # MAB348SP *OR* Rabbit mAb APP C-terminus, Y188, Epitomics Cat. # 1565-1, 1:5000; BACE1: Mouse mAb, 3D5 [32], 1:1000; C99 and C83: Rabbit mAb APP C-terminus, Y188, Epitomics Cat. # 1565-1, 1:5000; PS1: α-PS1NTF Rabbit pAb, a generous gift from Dr. Gopal Thinakaran (U. Chicago), 1:5000; Insulin Degrading Enzyme: α-IDE Rabbit pAb, Abcam Cat. # ab32216, 1:1000; Neprilysin: Anti-CD10 Rabbit mAb, EPR2997, Abcam Cat. # ab79423, 1:1000; Cleaved Caspase-3: Rabbit mAb, 5A1E, Cell Signaling Cat. # 9664, 1:1000; Caspase-3 (full-length): Rabbit pAb, Cell Signaling Cat. # 9662, 1:1000; p25 and p35: Rabbit pAb, C-19, Santa Cruz Biotechnology Cat. # sc-820, 1:1000) for 2 hrs at RT or overnight at 4°C. Blots were washed in TBST and incubated for 1 h in horseradish peroxidase (HRP)-conjugated goat anti rabbit (Jackson ImmunoResearch Laboratories, West Grove, PA) or horse anti-mouse (Vector Laboratories) secondary antibodies diluted 1:10,000 in 5% milk in TBST. Immunosignals were detected using enhanced chemiluminescence (EMD Millipore Luminata Classico, Crescendo, or Forte) and quantified using a Kodak Image Station 4000R imager (Rochester, NY). Densitometric analyses of immunoblots and images of Ponceau S-stained blots were performed using Kodak Molecular Imaging Software SE. Immunosignals were normalized to the measured intensity of whole lane Ponceau S staining. Values were expressed as percentages of the mean of the control.

### Immunofluorescence microscopy of tissue sections

Coronal sections of 30 μm were cut on a freezing sliding microtome and were selected with equivalent rostral-caudal locations using anatomical landmarks and the size and shape of the hippocampus and ventricles. Free-floating sections were washed 3× (10 min each) in TBS + 0.25% Triton-× 100 (TBS + T) and blocked for 90 min in 5% goat serum with TBS + T. Then they were washed 2× (10 min each) in 1% BSA with TBS + T before being incubated in anti-Aβ42 C-terminus specific rabbit polyclonal antibody (Invitrogen, Cat. No. 44-344, 1:1000) solution at 4°C overnight on an orbital shaker.

The sections were washed again the next day 2× (each 10 min) in 1% BSA with TBS + T before being incubated in secondary antibody solution of Alexa-Fluor Donkey anti rabbit-488 (A21206 along with DAPI (300nM) and thiazine red (1:60 k). Sections were washed in TBS in a dark room before mounting with ProLong gold antifade reagent (Life Technologies #P36934) and coverslipping #1.5 (VWR). Sections were imaged with a 10× air objective of a Keyence BZ-9000 Series microscope. Images were stitched using Keyence proprietary software. High magnification images of plaques were acquired on UV LSM510 laser scanning confocal microscope using laser lines of 405 nm (blue) 488 nm (green) and 561 nm (red).

### Amyloid plaque density

The total number of Aβ42-positive plaques was counted manually from one coronal section per mouse taken from the same mid-rostral-caudal location in the brain. 10-12 mice of each genotype (APP/Ld monogenic or D257A; APP/Ld bigenic) were used for plaque counting. The plaque density was then determined by dividing the total number of Aβ42-positive plaques by the total area of the section analyzed per mouse. The mean number of Aβ42-positive plaques per cm^2^ was then calculated for a given genotype. Additionally, mean plaque density was normalized to transgenic human APP protein level by dividing the plaque density of each mouse by that mouse’s respective NF value (see Human Aβ42 ELISA methods). SEMs and p-values were determined using the two-tailed Student’s t-test.

### Histology and related measurements

Coronal sections with equivalent mid-rostral-caudal locations in the brain were selected for histological staining and all related measurements. Hematoxylin staining was performed according to the manufacturer’s protocol (Vector Labs). Sections were dried on Superfrost Plus micro glass slides (VWR) for ~10 minutes to facilitate adhesion. Once stuck to the slide, the sections were then rehydrated with a few drops of water. The sections were then stained with Vector Hematoxylin QS for about 1 minute, and then rinsed with tap water until water washed over the sections was colorless. Afterward, sections were dehydrated in a series of alcohols (70%, 95%, 95%, 100%, and 100%), cleared in xylene, and coverslipped with Permount mounting medium (Fisher Scientific, SP15).

On hematoxylin stained sections, lateral cortical thickness was measured by the length of a line running perpendicular to the cortical layers connected from the tip of the CA1 region to the visible boundary of the cortex. Higher magnification images of the cortex stained with hematoxylin was acquired on Leica M165 FC or Zeiss Axioskop/CRi Nuance camera microscopes. Using images acquired on a Nikon Eclipse E800 microscope, the hippocampal area was measured by drawing a freehand selection around the hippocampus in ImageJ software (NIH).

Neuron density (number of neurons per mm^2^) was determined by counting the number of neurons in middle layers of the dorsal cortex in 30 μm thick hematoxylin stained coronal brain sections. Three sections spaced 15 sections apart that had the same relative rostral-caudal positions (centered over the mid-hippocampus; Figure 5A) in each mouse were chosen and two representative fields per section were counted using a Zeiss Axioskop microscope with a 100× oil objective in bright-field. All neurons (as identified by their large round nuclei) were counted in each 100× field, while glial cells (as identified by their small dense nuclei) were not counted. A total of 5 mice per genotype were counted and the mean neuron number per genotype was determined and converted to neuron density.

The Vector Laboratories (Burlingame, CA) ABC kit was used with DAB as chromogen to visualize the reaction product of cleaved caspase-3. On Day 1 of staining, sections were first incubated in 0.4% Triton at room temperature for 30 min. The sections were briefly rinsed in PBS before and after each incubation step, unless otherwise specified. The sections were subsequently incubated in vehicle/Serum (0.1% Triton X-100, 3% Goat Serum in PBS) at 4°C on shaker for 1 h. Sections were then incubated in H_2_O_2_ (1% in PBS) at 4°C for 1 h, followed by incubation in the primary antibody (anti-cleaved caspase 3, Rabbit mAb, 5A1E, 1:500), diluted in 1% BSA in TBS with 0.25% Triton X-100. On Day 2, sections were incubated in secondary antibody (Vector, Goat anti-Rabbit-HRP, 1:500) at 4°C for 2 hrs, followed by incubation in Reagent A + B (1% of each reagent in Vehicle solution) at 4°C for 2 hrs. The reagent solution was made at least 30 minutes prior to use. The sections were submerged in urea (120 mg/ml diluted in H_2_O)—no shaking—then briefly rinsed 2× in PBS followed by 3× in 0.05 M TRIS. Two development solutions (0.07% H_2_O_2_ in H_2_O; 1 mg/ml Diaminobenzidine in 0.05 M TRIS) were made and then mixed just before the development step. Sections were incubated in the development solution from 30 sec to 5 min until sufficient degree of color development occurred by visually inspecting the sections under a microscope. Finally, the sections were briefly rinsed in 0.05 M TRIS, dried overnight, dehydrated in a series of alcohols, cleared in xylene and coverslipped with Permount mounting medium.

### Statistical analysis

Data are presented as means and standard errors of the mean (SEMs, represented by error bars in histograms). N-values are stated in figure legends. GraphPad Prism (GraphPad Software, Inc., San Diego, CA) was used for all statistical analysis. The statistical significance between means of experimental and control groups was determined using the two-tailed Student’s t-test. For comparisons involving more than 2 groups, one-way ANOVA was used followed by post hoc Newman-Keuls multiple comparison test (*p < 0.05, **p < 0.01, ***p < 0.001).

## Abbreviations

Aβ: β-amyloid; AD: Alzheimer’s disease; APP: Amyloid precursor protein; BACE1: β-site amyloid precursor protein cleaving enzyme 1; PolgA: Gene for mitochondrial DNA polymerase γ catalytic core; APP/Ld: APP [V717I] London; IDE: Insulin degrading enzyme; NEP: Neprilysin; PS1: Presenilin 1; CTF: C-terminal fragment.

## Competing interests

The authors declare that they have no competing interests.

## Authors’ contributions

LK bred the mice, performed the experiments, and wrote the manuscript. RV conceived of the study, participated in its design and coordination, and edited the manuscript. GCK and TAP provided *PolgA*^*D257A*/*D257A*^ mitochondrial DNA mutator mice, and FVL provided APPV717I (APP/Ld) mice. All authors participated in the interpretation of results, read and approved the final manuscript.

## Supplementary Material

Additional file 1: Figure S1 *PolgA* D257A mutation does not affect endogenous mouse APP protein levels. (A) Brain homogenates were prepared from D257A; APP/Ld, D257A, APP/Ld, and wild type mice and 10 μg total protein per lane was randomly loaded and subjected to immunoblot analysis using anti-APP N-terminal antibody (22C11). Immunoblot signals were normalized to Ponceau S staining intensities in a given lane. The numbers on immunoblot correspond to a mouse with a specific genotype as denoted in the legend key (box). (B) APP immunosignals in (A) were measured and expressed as percentage of mean wild-type APP level. Note that endogenous APP levels were not significantly different between wild-type and D257A mice (mean ± SEM; **p < 0.01; Student’s t-test). Click here for file
