# Supplementary figures and images for: Increased mtDNA mutations with aging promotes amyloid accumulation and brain atrophy in the APP/Ld transgenic mouse model of Alzheimer’s disease
Source: Mol Neurodegener. 2014 May 2;9:16. doi: 10.1186/1750-1326-9-16 (PMC4028006; doi:10.1186/1750-1326-9-16)

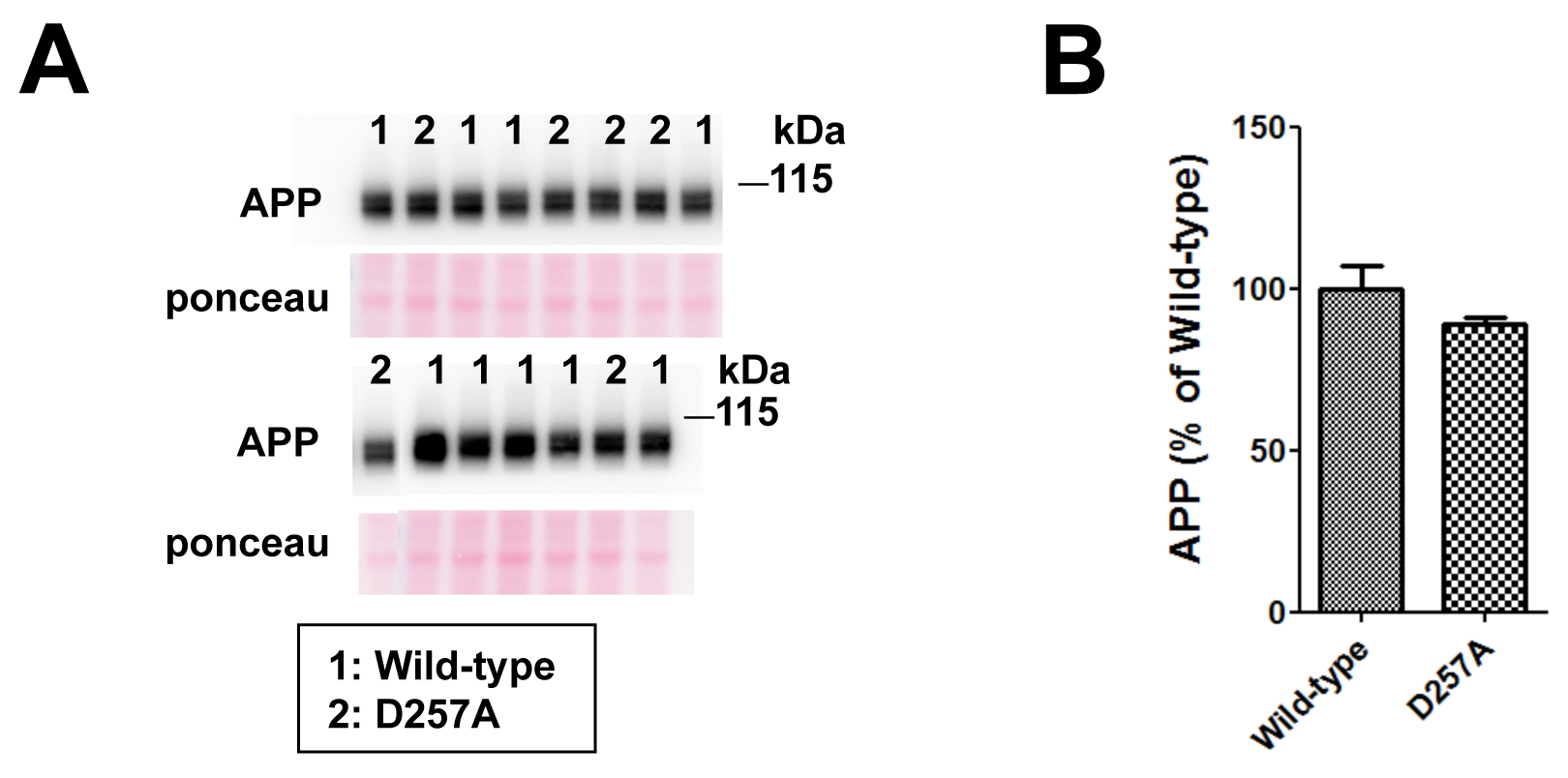

Supplement: Additional file 1: Figure S1 — PolgA D257A mutation does not affect endogenous mouse APP protein levels. (A) Brain homogenates were prepared from D257A; APP/Ld, D257A, APP/Ld, and wild type mice and 10 μg total protein per lane was randomly loaded and subjected to immunoblot analysis using anti-APP N-terminal antibody (22C11). Immunoblot signals were normalized to Ponceau S staining intensities in a given lane. The numbers on immunoblot correspond to a mouse with a specific genotype as denoted in the legend key (box). (B) APP immunosignals in (A) were measured and expressed as percentage of mean wild-type APP level. Note that endogenous APP levels were not significantly different between wild-type and D257A mice (mean ± SEM; **p < 0.01; Student’s t-test). [file 1750-1326-9-16-S1.tiff]
